# Prognostic Value of Immunonutritional Status and Radiologic Muscle Mass in Older Patients Receiving Immune Checkpoint Inhibitors: A Real-World Study

**DOI:** 10.3390/curroncol33080480

**Published:** 2026-08-14

**Authors:** Hülya Ertaş, Burcu Caner, Sibel Oyucu Orhan, Buket Şahin Çelik, Işıl Yüceışık, Yasemin Koç

**Affiliations:** 1 Medical Oncology, Ali Osman Sonmez Oncology Hospital, Bursa 16000, Turkey; 2 Medical Oncology, Ataturk State Hospital, Aydin 09020, Turkey; 3 Medical Oncology, Bursa City Hospital, Bursa 16110, Turkey; 4 Radiology, Bursa City Hospital, Bursa 16110, Turkey; 5 Department of Biostatistics, Institute of Health Sciences, Ankara University, Ankara 06230, Turkey

**Keywords:** immunotherapy, older adults, sarcopenia, Prognostic Nutritional Index, immune-related adverse events

## Abstract

Background: Identifying biomarkers for biological vulnerability in older patients with cancer remains challenging. We evaluated psoas muscle index (PMI) and immunonutritional indices’ association with toxicity and overall survival (OS) in patients ≥ 65 years receiving immune checkpoint inhibitors (ICIs). Methods: This retrospective study included 104 patients. Baseline sarcopenia was assessed via CT-derived PMI at the L3 level. Immunonutritional status was evaluated using the Prognostic Nutritional Index (PNI). Primary endpoint was immune-related adverse event (irAE) development; secondary endpoint was OS. Results: Median age was 71 years. Patients developing irAEs had significantly higher baseline PNI than those without (49.0 vs. 46.0, *p* = 0.042). OS did not differ significantly by irAE status (*p* = 0.859) or PMI group (*p* = 0.664). However, low PNI was strongly associated with poorer OS (17.0 months vs. not reached, *p* < 0.001). In a multivariable Cox model stratified by ICI treatment type, higher PNI remained independently associated with better OS (adjusted HR: 0.86, 95% CI: 0.81–0.93, *p* < 0.001). PNI correlated negatively with inflammatory markers NLR and SII. Conclusions: Baseline PNI was independently associated with OS, whereas PMI was not significantly associated with survival in this cohort. These findings support the potential prognostic utility of PNI in older patients receiving immunotherapy but should not be interpreted as demonstrating superiority over comprehensive sarcopenia assessment.

## Simple Summary

Older adults receiving immune checkpoint inhibitors (ICIs) represent a highly heterogeneous population, and predicting treatment outcomes remains challenging. Although loss of muscle mass (sarcopenia) has been proposed as a marker of frailty, it may not fully reflect the complex interaction between nutrition, inflammation, and immune function. In this real-world retrospective study, we evaluated radiologic sarcopenia using the psoas muscle index (PMI) and immunonutritional status using the Prognostic Nutritional Index (PNI) in 104 patients aged 65 years or older treated with ICIs. We found that patients with a higher baseline PNI were more likely to develop immune-related adverse events, while low PNI was strongly associated with significantly shorter overall survival. In contrast, PMI was not associated with treatment toxicity or survival. These findings suggest that PNI showed a stronger association with survival than PMI in this cohort. However, because PMI reflects muscle quantity rather than muscle function, these results should not be interpreted as demonstrating the general superiority of immunonutritional assessment over comprehensive sarcopenia assessment. Because PNI is calculated from routine laboratory tests, it is an inexpensive and easily accessible biomarker that may help identify vulnerable patients and support individualized treatment decisions. Larger prospective studies are needed to confirm these findings.

## 1. Introduction

The introduction of immune checkpoint inhibitors (ICIs) has fundamentally changed the management of advanced solid malignancies by providing durable clinical responses and prolonged survival in a wide range of tumor types, particularly non-small-cell lung cancer (NSCLC), renal cell carcinoma, melanoma, and urothelial carcinoma [1,2]. As indications for ICIs continue to expand, an increasing proportion of patients receiving immunotherapy are older adults [3]. Since cancer incidence rises markedly with age, individuals aged 65 years and older now represent a substantial proportion of patients considered for immune checkpoint blockade. Although chronological age alone should not preclude immunotherapy, treatment outcomes among older patients remain highly variable, emphasizing the need for biomarkers that more accurately reflect biological fitness than age itself.

The biological processes associated with aging introduce unique challenges in cancer immunotherapy. Age-related remodeling of the immune system, commonly referred to as immunosenescence, is characterized by impaired adaptive immune responses, reduced T-cell diversity, altered cytokine production, and diminished immune surveillance [4,5]. At the same time, older adults frequently develop a chronic low-grade inflammatory state, known as inflammaging, which has been associated with frailty, metabolic dysregulation, and adverse oncologic outcomes. These age-related changes may influence both the antitumor activity of ICIs and the development of immune-related adverse events (irAEs), making therapeutic responses less predictable than in younger populations [6].

In routine oncology practice, treatment decisions are generally guided by clinical parameters such as Eastern Cooperative Oncology Group (ECOG) performance status, body mass index (BMI), chronological age, and comorbidity burden. However, these conventional measures often fail to capture the multidimensional physiological alterations accompanying aging, including declining nutritional reserve, progressive muscle loss, systemic inflammation, and impaired immune competence. Consequently, there is growing interest in identifying practical biomarkers that more accurately reflect biological ageing and improve risk stratification, thereby facilitating individualized treatment strategies, optimizing patient selection, and ultimately improving both survival outcomes and treatment tolerability.

Frailty has emerged as one of the most important determinants of treatment tolerance and survival in older patients with cancer [7]. Unlike chronological age, frailty reflects cumulative declines across multiple physiological systems, resulting in diminished functional reserve and increased vulnerability to external stressors, including systemic anticancer therapy. Growing evidence indicates that frailty adversely affects treatment completion, hospitalization, toxicity, quality of life, and overall survival in patients receiving immunotherapy [8]. Consequently, contemporary geriatric oncology guidelines recommend integrating frailty assessment into therapeutic decision-making whenever feasible, although comprehensive geriatric assessment remains resource-intensive and is not routinely implemented in many oncology centers.

Among the biological manifestations of frailty, sarcopenia has received considerable attention as a potential prognostic biomarker in oncology. Defined by progressive loss of skeletal muscle mass and function, sarcopenia is highly prevalent in older adults and is further aggravated by cancer-associated inflammation, malnutrition, and physical inactivity. Computed tomography (CT)-based body composition analysis has become the reference method for objective assessment of skeletal muscle because it is readily available in routine oncologic practice and provides reproducible quantitative measurements without additional patient burden. Various CT-derived indices, including the skeletal muscle index (SMI), psoas muscle index (PMI), and measures of muscle radiodensity reflecting myosteatosis, have been investigated as imaging biomarkers of biological aging and treatment vulnerability [9,10,11].

Several systematic reviews and meta-analyses have demonstrated that low skeletal muscle mass is associated with inferior response rates, shorter progression-free survival, reduced overall survival, and an increased risk of treatment-related toxicity in patients receiving immune checkpoint inhibitors [12,13,14]. Nevertheless, most available studies have included heterogeneous adult populations, whereas evidence specifically focusing on older patients remains limited. Furthermore, considerable methodological heterogeneity persists regarding the definition of sarcopenia, anatomical measurement sites, and threshold values, making direct comparisons across studies challenging and limiting the clinical applicability of CT-derived muscle parameters alone.

Because cancer progression, immune competence, systemic inflammation, and nutritional status are closely interconnected, increasing attention has shifted toward immunonutritional biomarkers that integrate these biological processes [15]. Among these, the Prognostic Nutritional Index (PNI), derived from serum albumin concentration and peripheral lymphocyte count, has emerged as a simple, inexpensive, and reproducible indicator of host nutritional reserve and immune function. Unlike radiologic measures that primarily quantify muscle quantity, PNI may better reflect the dynamic interaction between nutritional status, systemic inflammation, and antitumor immunity, and provide a more comprehensive assessment of host condition, making PNI an attractive biomarker for patients undergoing immune checkpoint blockade [16].

Growing evidence suggests that a low baseline PNI is associated with poorer survival and reduced treatment efficacy across several malignancies, including patients receiving immune checkpoint inhibitors [17]. Compared with imaging-based measures of muscle quantity, PNI reflects dynamic physiological processes that may directly influence immune activation, treatment response, and tolerance to immunotherapy. Moreover, as it is derived from routinely available laboratory parameters, PNI is inexpensive, objective, and easily incorporated into everyday clinical practice without additional imaging or specialized software.

Despite these promising findings, the relative prognostic value of immunonutritional status compared with radiological sarcopenia remains uncertain, particularly in older adults. Most previous studies have evaluated either CT-derived muscle parameters or laboratory-based biomarkers separately, while direct comparisons between these approaches are scarce. In addition, available evidence largely originates from heterogeneous cohorts including different age groups, tumour types, and treatment strategies, limiting its applicability to geriatric oncology. Therefore, whether immunonutritional assessment provides prognostic information beyond CT-derived skeletal muscle measurements in older patients treated with immune checkpoint inhibitors remains an important unanswered clinical question.

To address this knowledge gap, we conducted a retrospective study evaluating patients aged 65 years and older who received immune checkpoint inhibitor-based therapy. We compared the prognostic significance of the Prognostic Nutritional Index (PNI) and the computed tomography-derived Psoas Muscle Index (PMI) for treatment-related toxicity and overall survival. We hypothesized that PNI would provide clinically relevant prognostic information alongside radiological assessment of skeletal muscle and could represent a practical biomarker for routine geriatric oncology practice.

## 2. Materials and Methods

### 2.1. Study Design and Patient Population

This retrospective, single-center study was conducted at a tertiary oncology center and included 104 patients aged ≥65 years who received immune checkpoint inhibitor (ICI)-based therapy for advanced malignancies. The primary survival analyses were performed in the overall study cohort. Given the heterogeneity of tumor types and the predominance of patients with lung cancer, an additional subgroup sensitivity analysis was performed to assess the robustness of the survival findings. Patients were eligible for inclusion if they:

1. Were aged 65 years or older at the initiation of immunotherapy;
2. Received at least one cycle of an ICI-based regimen;
3. Had available baseline laboratory parameters and contrast-enhanced computed tomography (CT) imaging performed within 30 days before treatment initiation.

Patients with incomplete clinical or laboratory records, unavailable baseline imaging, or inadequate radiologic quality for body composition analysis were excluded from the study.

The study was conducted in accordance with the principles of the Declaration of Helsinki and approved by the institutional ethics committee.

### 2.2. Radiological Assessment of Sarcopenia

Baseline CT images were retrospectively reviewed by experienced observers blinded to clinical outcomes. Skeletal muscle assessment was performed using axial CT images obtained at the level of the third lumbar vertebra (L3), a validated anatomical landmark for body composition analysis.

The cross-sectional areas of the bilateral psoas muscles were measured and normalized to height squared to calculate the Psoas Muscle Index (PMI, cm^2^/m^2^). In addition, mean psoas muscle attenuation values expressed in Hounsfield units (HU) were recorded as indicators of muscle quality and myosteatosis.

For comparative analyses, patients were stratified into low and high PMI groups according to the median PMI value of the study population.

### 2.3. Immunonutritional and Inflammatory Markers

Baseline laboratory parameters obtained prior to the first cycle of immunotherapy were used to calculate immunonutritional and systemic inflammatory indices.

The Prognostic Nutritional Index (PNI) was calculated using the following formula:

PNI = 10 × serum albumin (g/dL) + 0.005 × peripheral lymphocyte count (/mm^3^)

The Neutrophil-to-Lymphocyte Ratio (NLR) was calculated as:

NLR = absolute neutrophil count/absolute lymphocyte count

The Systemic Immune-Inflammation Index (SII) was calculated as:

SII = (platelet count × neutrophil count)/lymphocyte count

For Kaplan–Meier survival visualization, PNI was categorized into low and high groups according to the cohort median.

### 2.4. Comorbidity Assessment

Comorbidity burden was assessed using the age-adjusted Charlson Comorbidity Index (CCI). Higher CCI scores indicated greater comorbidity burden and reduced physiological reserve.

### 2.5. Outcome Definitions

The primary endpoint of the study was the development of immune-related adverse events (irAEs). Adverse events were graded according to the Common Terminology Criteria for Adverse Events (CTCAE) version 5.0.

Secondary endpoints included:

- Overall survival (OS);
- Severe treatment-related toxicity;
- Treatment discontinuation due to toxicity.

Severe toxicity was defined as Grade ≥ 3 treatment-related adverse events requiring hospitalization and/or systemic corticosteroid therapy.

Overall survival was defined as the interval between initiation of immunotherapy and death from any cause or last follow-up. Survival analyses were performed according to baseline PMI, PNI, and irAE status using Kaplan–Meier methods and Cox proportional hazards regression analyses.

### 2.6. Statistical Analysis

Statistical analyses were performed using IBM SPSS Statistics version 30.0 (IBM Corp., Armonk, NY, USA) and R version 4.5.1 (13 June 2025) Continuous variables were assessed for normality using the Shapiro–Wilk test and are presented as mean ± standard deviation or median (interquartile range), as appropriate. Categorical variables are presented as numbers and percentages. Between-group comparisons were performed using the independent-samples *t*-test or Mann–Whitney U test for continuous variables and the chi-square test or Fisher’s exact test for categorical variables, as appropriate.

Overall survival (OS) was defined as the time from initiation of immune checkpoint inhibitor therapy to death from any cause or the last follow-up. Kaplan–Meier analysis was used to estimate survival distributions, and differences between groups were assessed using the log-rank test. For Kaplan–Meier visualization, PNI and PMI were categorized as low or high according to their cohort median values. To avoid potential information loss associated with dichotomization, PNI and PMI were analyzed as continuous variables in Cox proportional hazards regression models. Hazard ratios (HRs) with 95% confidence intervals (CIs) were reported.

Variables considered clinically relevant and potential confounders were evaluated for inclusion in the multivariable Cox regression analysis. The final multivariable Cox regression model included PNI, age, and antibiotic use. Because ICI treatment type did not satisfy the proportional hazards assumption in the initial model, the final Cox model was stratified by ICI treatment type, allowing the baseline hazard to vary across ICI regimens without estimating a common hazard ratio for treatment type. PNI and age were entered as continuous variables. The proportional hazards assumption for the final stratified model was evaluated using Schoenfeld residuals. Steroid use was additionally evaluated but was not retained in the final model because its inclusion did not significantly improve model fit. ECOG performance status could not be incorporated into the adjusted analyses because this variable was not consistently available in the retrospective dataset.

Given the predominance of patients with lung cancer and the heterogeneity of tumor types in the overall cohort, a sensitivity analysis restricted to patients with lung cancer was additionally performed. In this subgroup, PNI and PMI were evaluated as continuous variables in univariate Cox regression analyses. Spearman correlation analysis was used to assess associations between PNI and clinical or inflammatory parameters. All tests were two-sided, and a *p* value < 0.05 was considered statistically significant.

## 3. Results

### 3.1. Patient Characteristics and Baseline Demographics

The study population comprised 104 older patients (median age 71 years) with various malignancies. Baseline clinical and laboratory characteristics, stratified by the occurrence of immune-related adverse events (irAEs), are detailed in Table 1. Overall, baseline parameters including age, comorbidity burden (CCI), and sarcopenia-related indices (PMI and psoas density) were comparable between patients who developed irAEs and those who did not.

Notably, patients who experienced irAEs exhibited significantly higher baseline PNI values than those without irAEs (49.0 vs. 46.0; *p* = 0.042).

Total CCI and PMI differed significantly across ICI treatment groups (*p* = 0.004 and *p* = 0.028, respectively). Descriptively, the anti–PD-L1 group had the lowest median CCI and the highest median PMI. The frequency of irAEs was numerically highest in the anti–PD-1 group, although the overall between-group difference did not reach statistical significance (*p* = 0.058). This features was shown in Table 2.

### 3.2. Survival Outcomes and Prognostic Factors

Kaplan–Meier survival analyses were performed to evaluate the association of irAE status, PMI, and PNI with overall survival.

**Figure 1:**
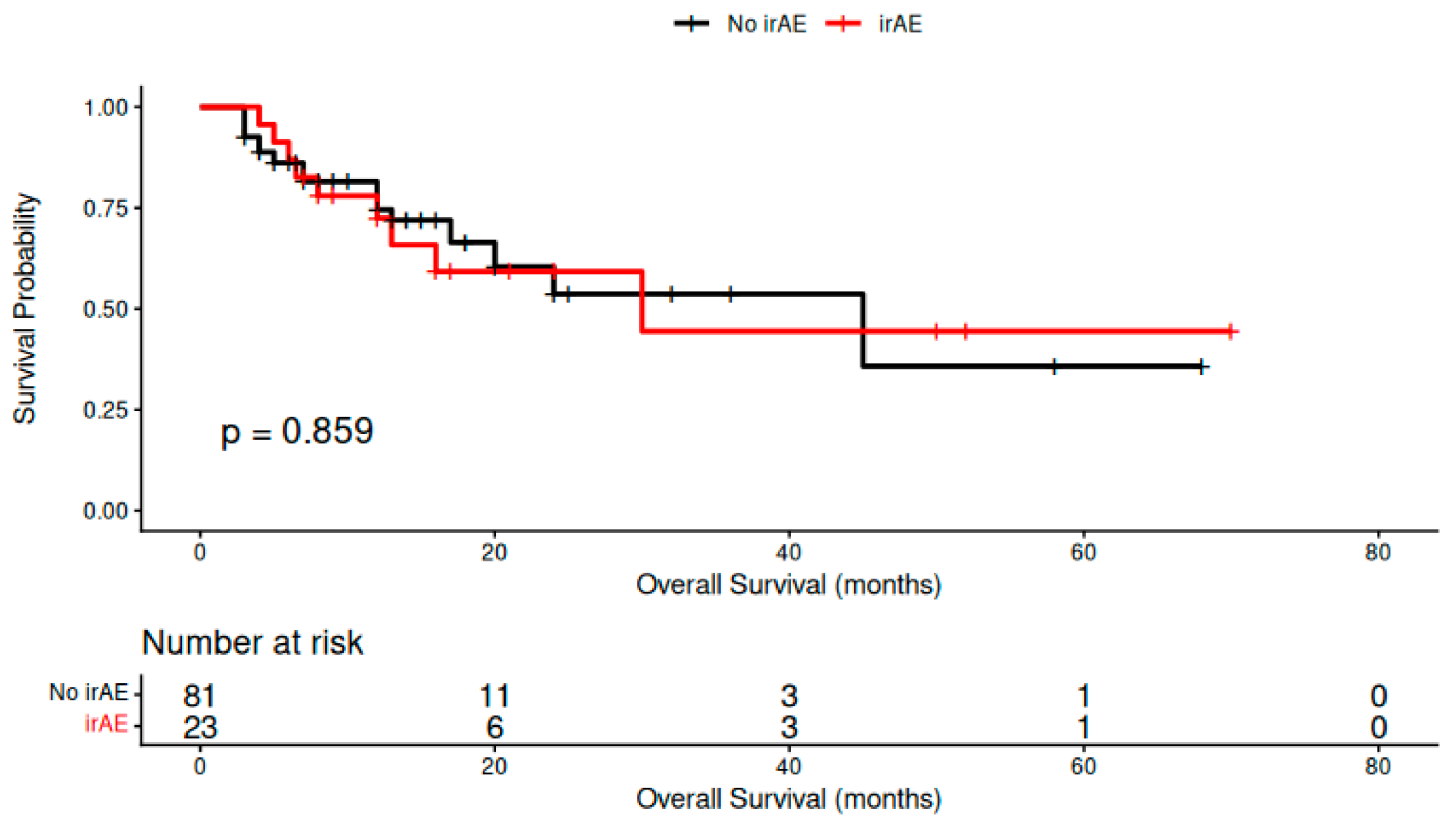
Kaplan–Meier curves for overall survival according to irAE status.

Kaplan–Meier survival analysis demonstrated no significant difference in overall survival between patients with and without irAEs (log-rank *p* = 0.859). Median overall survival was 45.0 months in patients without irAEs and 30.0 months in those who developed irAEs (Figure 1).

**Figure 2:**
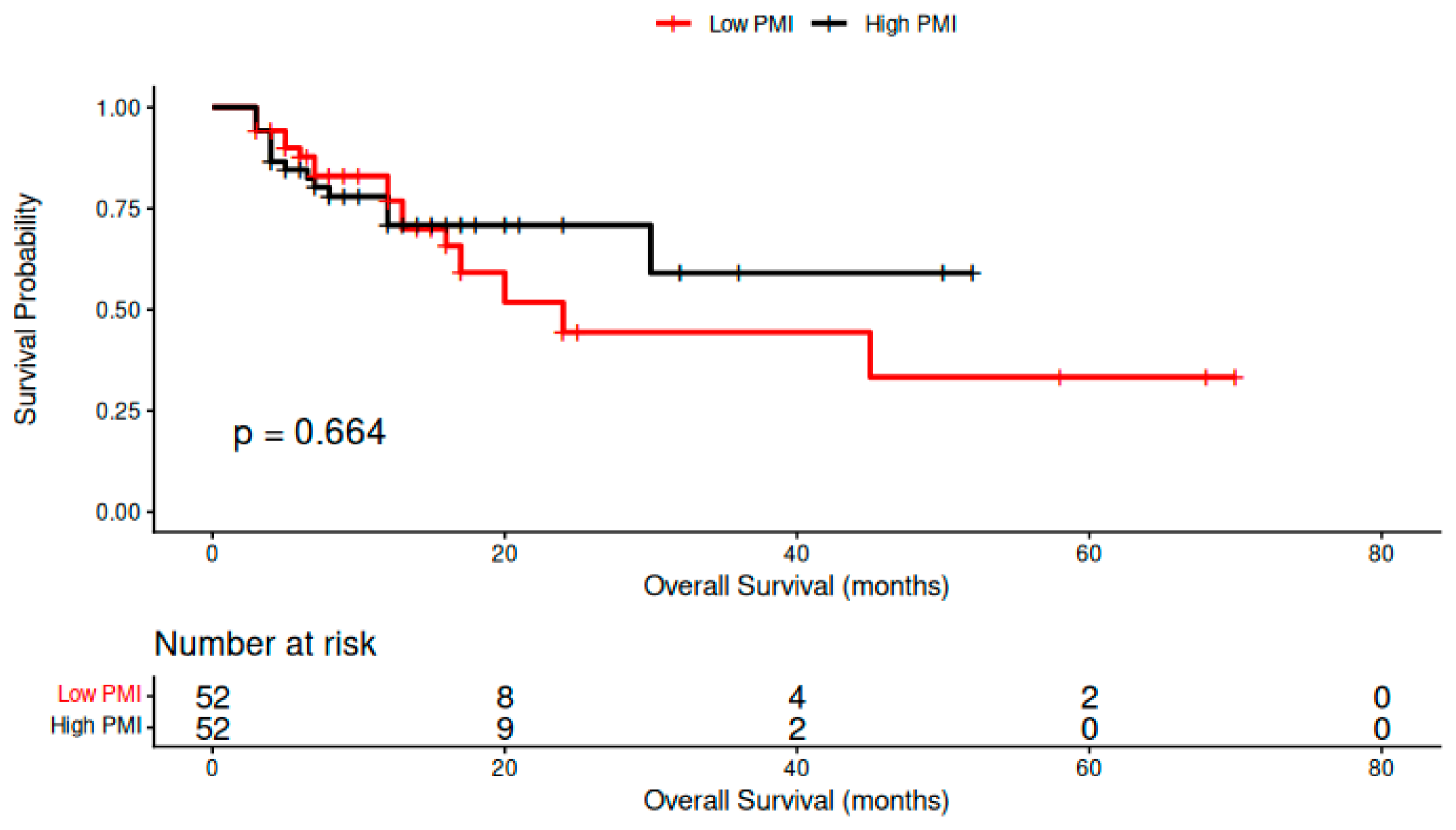
Kaplan–Meier curves for overall survival according to PMI group.

Kaplan–Meier survival analysis according to PMI group showed no statistically significant difference in overall survival between patients with low and high PMI values (log-rank *p* = 0.664). Median overall survival was 24.0 months in the low PMI group, whereas median overall survival was not reached in the high PMI group during follow-up (Figure 2).

**Figure 3:**
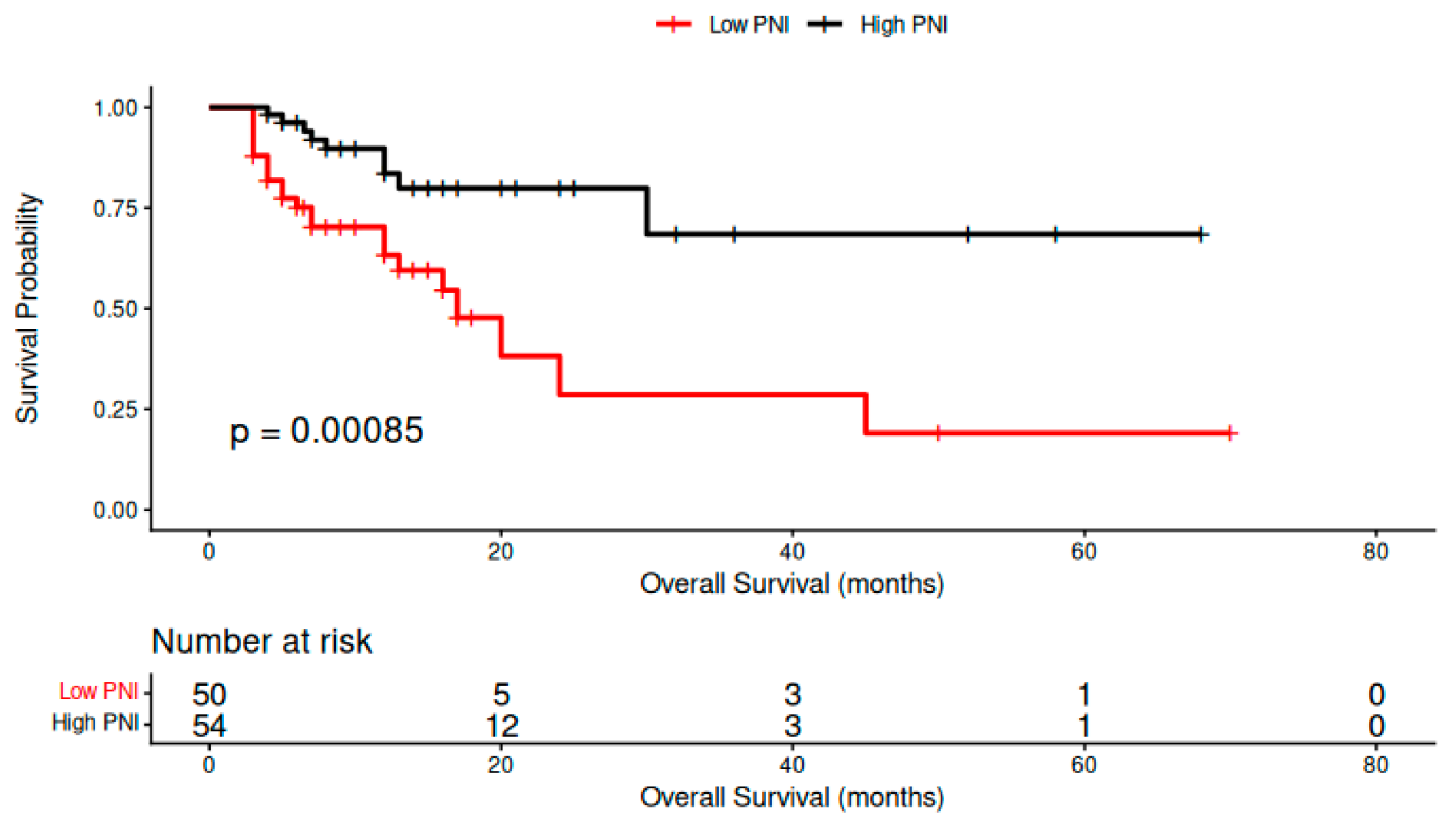
Kaplan–Meier curves for overall survival according to PNI group.

Kaplan–Meier survival analysis demonstrated significantly poorer overall survival in patients with low PNI values compared with those with high PNI values (log-rank *p* < 0.001) (Figure 3). Median overall survival was 17.0 months in the low PNI group, whereas median overall survival was not reached in the high PNI group during follow-up (Table 3).

In univariate Cox regression analysis, advanced age, higher total CCI score, and lower PNI were significantly associated with poorer overall survival. Each one-year increase in age was associated with an 8% increase in the mortality hazard (HR: 1.08, 95% CI: 1.00–1.16, *p* = 0.037), while each one-point increase in total CCI score was associated with a 32% increase in mortality hazard (HR: 1.32, 95% CI: 1.04–1.67, *p* = 0.022). In contrast, each one-unit increase in PNI was associated with a 12% reduction in mortality hazard (HR: 0.88, 95% CI: 0.82–0.94, *p* < 0.001). PMI was not significantly associated with overall survival when analyzed as a continuous variable (HR: 0.85, 95% CI: 0.62–1.16, *p* = 0.313). Similarly, irAE status was not significantly associated with mortality risk (HR: 1.07, 95% CI: 0.49–2.33, *p* = 0.874) (Table 4).

**Figure 4:**
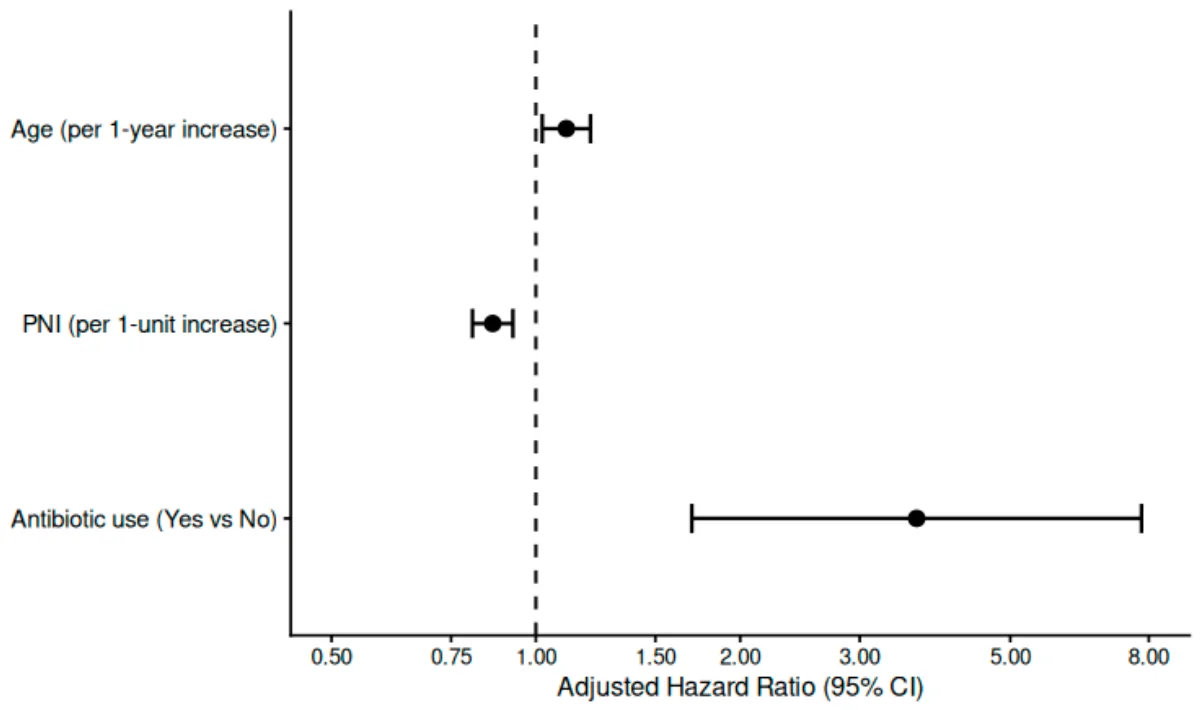
Forest plot of multivariable Cox regression analysis for overall survival. Forest plot showing adjusted hazard ratios for PNI, age, and antibiotic use from the Cox proportional hazards model stratified by ICI treatment type. HR, hazard ratio; CI, confidence interval; PNI, Prognostic Nutritional Index; ICI, immune checkpoint inhibitor.

In the multivariable Cox regression model stratified by ICI treatment type, higher PNI remained independently associated with better overall survival (adjusted HR: 0.86, 95% CI: 0.81–0.93, *p* < 0.001). Forest plot of multivariable Cox regression analysis for overall survival was shown with Figure 4. Increasing age was independently associated with higher mortality (adjusted HR: 1.11, 95% CI: 1.02–1.20, *p* = 0.013), as was antibiotic use (adjusted HR: 3.64, 95% CI: 1.70–7.80, *p* < 0.001). The proportional hazards assumption was satisfied for the stratified model (global Schoenfeld test, *p* = 0.37). This was shown in Table 5.

**Figure 5:**
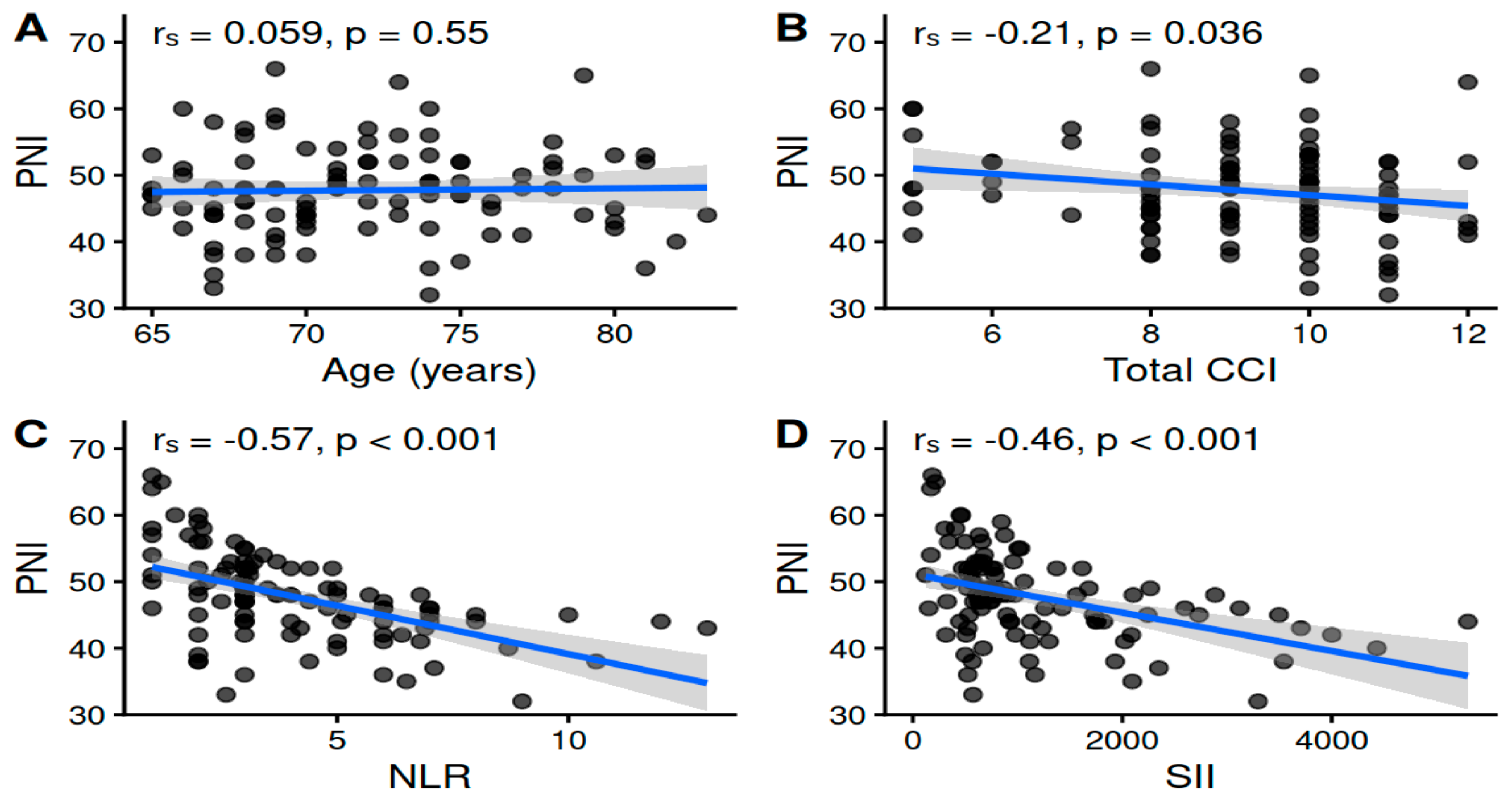
Correlations between PNI and clinical–inflammatory parameters. Scatter plots demonstrating correlations between PNI and age (**A**), total CCI score (**B**), NLR (**C**), and SII (**D**). Spearman correlation analysis demonstrated significant negative correlations of PNI with total CCI score, NLR, and SII, whereas no significant correlation was observed with age. Solid lines represent linear trend lines with 95% confidence intervals; correlation coefficients and *p* values were calculated using Spearman’s rank correlation.

Given the predominance of lung cancer in the study cohort, a sensitivity analysis was performed in patients with lung cancer (n = 87; 25 deaths). Consistent with the findings in the overall cohort, higher PNI was significantly associated with improved OS when analyzed as a continuous variable (HR: 0.83, 95% CI: 0.77–0.90, *p* < 0.001), whereas PMI was not significantly associated with OS (HR: 0.80, 95% CI: 0.56–1.13, *p* = 0.204). Correlations between PNI and clinical–inflammatory parameters was shown with Figure 5.

## 4. Discussion

In the present study, we evaluated the prognostic and predictive relevance of sarcopenic, inflammatory, and immunonutritional parameters in older patients receiving immune checkpoint inhibitor (ICI)-based therapy. The principal finding was that baseline Prognostic Nutritional Index (PNI) was independently associated with overall survival (OS), whereas Psoas Muscle Index (PMI) and the development of immune-related adverse events (irAEs) were not significantly associated with survival. In addition, patients who developed irAEs had significantly higher baseline PNI values. Collectively, these findings indicate that PNI showed a stronger association with survival than PMI in this cohort of older patients treated with ICIs. The increasing use of ICIs in older adults has highlighted the limitations of relying exclusively on chronological age, Eastern Cooperative Oncology Group (ECOG) performance status, body mass index, or comorbidity burden when estimating treatment tolerance and prognosis. Older patients with cancer constitute a biologically heterogeneous population with substantial variation in frailty, physiological reserve, nutritional status, immune competence, and age-related inflammatory activity. Recent geriatric oncology reviews and systematic assessments have therefore emphasized the importance of incorporating host-related and geriatric parameters into immunotherapy decision-making [1,2,3,4]. Nevertheless, no single conventional clinical measure fully captures the multidimensional vulnerability of older adults. Biomarkers that integrate nutritional reserve, systemic inflammation, and immune function may consequently offer additional prognostic information in this population.

Sarcopenia has been associated with adverse clinical outcomes across several malignancies and treatment modalities. In patients receiving ICIs, systematic reviews and meta-analyses have reported associations between low skeletal muscle mass and shorter progression-free survival and OS, although the magnitude and consistency of these associations vary considerably among studies [17,18,19]. This heterogeneity may be attributable to differences in tumour type, treatment line, CT measurement level, sarcopenia definition, cut-off values, and adjustment for clinical confounders. In addition, radiological assessments of muscle quantity do not directly evaluate muscle strength or physical performance, which are central components of contemporary sarcopenia definitions.

In our cohort, PMI was not significantly associated with OS or irAE development. Although patients with low PMI had numerically shorter survival, this difference did not reach statistical significance. Several explanations should be considered. First, PMI represents the cross-sectional area of a relatively small muscle group and may not fully reflect whole-body skeletal muscle reserve. Second, a single baseline measurement cannot capture subsequent changes in muscle mass during cancer progression or treatment. Third, muscle quantity alone may inadequately represent the broader biological construct of frailty, which also includes functional decline, nutritional depletion, comorbidities, systemic inflammation, and impaired physiological resilience. Accordingly, the absence of a statistically significant association between PMI and survival in our study should not be interpreted as evidence that body composition is clinically irrelevant, but rather that an isolated psoas-based measurement may have limited discriminatory value in a heterogeneous geriatric immunotherapy population.

In contrast, baseline PNI was independently associated with OS. PNI combines serum albumin concentration and peripheral lymphocyte count and therefore reflects two clinically relevant dimensions of host condition: nutritional and inflammatory reserve and immune competence. Serum albumin is influenced not only by nutritional intake but also by systemic inflammation, hepatic synthesis, disease burden, and cancer-associated catabolism. Peripheral lymphocyte count, meanwhile, may partly reflect the capacity of the host immune system to mount and maintain an antitumour response. Thus, PNI may capture biological processes that are not represented by muscle quantity alone.

Our results are consistent with the available immunotherapy literature. A systematic review and meta-analysis of 12 studies including 1359 patients treated with ICIs found that a low pretreatment PNI was associated with poorer objective response, lower disease control, shorter progression-free survival, and inferior OS [16]. More recent tumour-specific studies have similarly supported the prognostic relevance of pretreatment PNI in patients receiving ICI-based therapy [20,21]. The association observed after multivariable adjustment extends these findings to an older, real-world population and supports the potential clinical utility of PNI as an inexpensive and readily available biomarker.

Another notable finding was that patients who developed irAEs had higher baseline PNI values. A recent study by Furuno et al. also investigated pretreatment PNI as a potential predictor of irAE occurrence and reported an association between host immunonutritional status and subsequent immune toxicity [22]. This relationship is biologically plausible. Patients with preserved nutritional status and higher circulating lymphocyte levels may possess greater immunological capacity for T-cell activation following checkpoint inhibition, potentially facilitating both antitumour immunity and immune-mediated toxicity. Nevertheless, this interpretation remains hypothesis-generating. PNI is a nonspecific composite marker and does not directly measure T-cell function, cytokine activity, immune repertoire diversity, or the tumour immune microenvironment.

In the present study, irAE development itself was not significantly associated with OS. This finding differs from several retrospective studies and meta-analyses reporting improved outcomes among patients who experience irAEs. For example, a large 2024 cohort study in metastatic NSCLC found that the occurrence and severity of irAEs were associated with survival, although the relationship was influenced by the type and grade of toxicity [23]. Other studies have likewise reported positive associations between irAEs and ICI efficacy. However, these analyses are vulnerable to immortal-time bias, treatment-duration bias, differences in irAE ascertainment, and the confounding effects of corticosteroid exposure and treatment discontinuation. Patients must remain alive and on treatment long enough to develop an irAE, which may artificially create an apparent survival advantage. In older adults, the clinical impact of irAEs may also differ according to frailty, organ reserve, comorbidity burden, toxicity severity, and the ability to tolerate immunosuppressive treatment. Recent reviews therefore conclude that the prognostic implications of irAEs in geriatric oncology remain incompletely defined [24].

The finding that higher PNI was associated with irAE occurrence, whereas irAEs themselves were not associated with OS, may indicate that baseline host condition is more prognostically informative than toxicity development alone. Preserved immunonutritional status may increase the likelihood of measurable immune activation, but the survival consequences of an irAE probably depend on multiple subsequent factors, including toxicity grade, affected organ system, treatment interruption, corticosteroid exposure, tumour response, and competing mortality risks. The association observed in our study should therefore not be interpreted as evidence that irAEs are desirable or that higher PNI inevitably predicts clinically beneficial immune activation.

Comorbidity burden, assessed using the Charlson Comorbidity Index, was significantly associated with mortality in the univariate analysis. However, CCI was not included in the final multivariable model because the index used in this study was age-adjusted, while chronological age was modeled separately as a continuous covariate. Therefore, the independent prognostic contribution of CCI beyond the other variables included in the final model was not evaluated.

Taken together, our findings suggest that PNI may provide clinically relevant prognostic information beyond that obtained from an isolated CT-based PMI measurement in older patients receiving ICI-based therapy. Because PNI is objective, inexpensive, reproducible, and routinely available, it may represent a practical adjunct to conventional clinical and geriatric assessment. However, PNI should not be regarded as a substitute for comprehensive geriatric assessment, functional testing, or validated frailty screening. Rather, it may serve as an accessible first-line marker for identifying patients who require more detailed nutritional, functional, or geriatric evaluation.

### Clinical Implications and Limitations

The results of this study indicate that PNI showed a stronger association with overall survival than PMI in this cohort of older patients treated with immune checkpoint inhibitors. Because PNI is derived from routinely available laboratory parameters, it offers a practical, low-cost, and easily applicable tool that could support risk stratification in everyday clinical practice.

This study has several limitations that should be considered when interpreting the findings. Its retrospective design and relatively small sample size may have influenced the robustness of the analyses. Furthermore, the limited number of deaths may have reduced the precision of some effect estimates; therefore, the adjusted findings should be interpreted cautiously and validated in larger cohorts. In addition, sarcopenia was evaluated solely using CT-based muscle measurements, without complementary assessments of muscle strength or physical performance, such as handgrip strength or gait speed. ECOG performance status could not be incorporated into the adjusted survival analyses because it was not consistently available in the retrospective dataset; therefore, residual confounding by functional status cannot be excluded. The inclusion of patients with different tumor types may limit the generalizability of the overall-cohort findings. To address this heterogeneity, an additional sensitivity analysis was performed in the predominant lung cancer subgroup, in which the association between higher PNI and improved OS remained consistent. Nevertheless, this subgroup analysis does not fully eliminate the potential influence of tumor-specific prognostic factors. Despite these limitations, our findings provide real-world evidence supporting the prognostic value of immunonutritional status in older patients receiving immunotherapy and highlight the potential clinical utility of combining nutritional and radiologic assessments in this setting.

## 5. Conclusions

Baseline PNI was independently associated with overall survival in older patients treated with ICIs, whereas PMI was not significantly associated with survival in this cohort. These findings support PNI as a potentially useful and readily available prognostic biomarker; however, they do not establish its superiority over comprehensive sarcopenia assessment. Prospective multicenter studies incorporating functional measures and comprehensive geriatric assessment are warranted to validate these findings.

## Figures and Tables

**Table 1:** Baseline characteristics according to irAE status.

Variable	No irAE (n = 81)	irAE (n = 23)	*p* Value
Age, years	71.00 (7)	74.00 (6)	0.213
Total CCI	9.00 (2)	10.00 (3)	0.355
PMI	2.35 (2.00)	2.62 (0.97)	0.716
Mean psoas density	25.50 (15.8)	28.50 (12.5)	0.508
Albumin	4.00 (0.60)	4.00 (0.30)	0.482
NLR	3.20 (3.5)	3.00 (2.4)	0.218
SII	809.00 (1170)	739.00 (450)	0.387
PNI	46.00 (10)	49.00 (5)	0.042
Gender, n (%)			
Male	69 (85.2%)	19 (82.6%)	0.762
Female	12 (14.8%)	4 (17.4%)	
Steroid use	9 (11.1%)	5 (21.7%)	0.188
Antibiotic use	16 (19.8%)	8 (34.8%)	0.131
PPI use	24 (29.6%)	10 (43.5%)	0.211
ICI type			0.058
Anti–PD-1	27 (33.3%)	14 (60.9%)	
Anti–PD-L1	17 (21.0%)	3 (13.0%)	
Combination	37 (45.7%)	6 (26.1%)	

Data are presented as median (IQR) or n (%). Continuous variables were compared using the Mann–Whitney U test, and categorical variables using the chi-square or Fisher’s exact test.

**Table 2:** Baseline characteristics according to ICI regimen.

Variable	Anti-PD-1 (n = 41)	Anti-PD-L1 (n = 20)	Combination (n = 43)	*p*-Value
Age, years	73.0 (9)	71.5 (6)	71.0 (7)	0.872
Total CCI	9.0 (2)	7.5 (4)	10.0 (3)	**0.004**
Mean psoas density	27.5 (14.0)	22.5 (20.3)	28.0 (11.5)	0.443
PMI	2.15 (0.95)	2.83 (2.01)	2.62 (2.33)	**0.028**
NLR	3.0 (2.2)	3.85 (2.0)	4.2 (3.4)	0.081
Albumin	4.0 (0.35)	4.1 (0.38)	3.9 (0.60)	0.418
SII	668 (715)	833 (360)	1007 (1520)	0.067
PNI	48.0 (8)	48.0 (8)	46.0 (10)	0.296
Male sex, n (%)	33 (80.5%)	20 (100%)	35 (81.4%)	0.105
Steroid use, n (%)	3 (7.3%)	4 (20.0%)	7 (16.3%)	0.308
Antibiotic use, n (%)	7 (17.1%)	7 (35.0%)	10 (23.3%)	0.296
PPI use, n (%)	8 (19.5%)	9 (45.0%)	17 (39.5%)	0.063
irAE development, n (%)	14 (34.1%)	3 (15.0%)	6 (14.0%)	0.058
Exitus, n (%)	15 (36.6%)	2 (10.0%)	14 (32.6%)	0.090

Data are presented as median (IQR) or n (%). Continuous variables were compared using the Kruskal–Wallis test and categorical variables using the chi-square test. Statistically significant *p*-values are shown in bold.

**Table 3:** Kaplan–Meier survival analysis summary.

Variable	Median OS (Months)	Log-Rank *p*-Value
irAE absent	45.0	0.859
irAE present	30.0	
Low PMI	24.0	0.664
High PMI	NR	
Low PNI	17.0	<0.001
High PNI	NR	

NR: not reached.

**Table 4:** Univariate Cox regression analysis for overall survival.

Variable	HR	95% CI	*p* Value
Age, per 1-year increase	1.08	1.00–1.16	0.037
Total CCI, per 1-point increase	1.32	1.04–1.67	0.022
PMI, per 1-unit increase	0.85	0.62–1.16	0.313
PNI, per 1-unit increase	0.88	0.82–0.94	<0.001
irAE status	1.07	0.49–2.33	0.874

Age, total CCI, PMI, and PNI were analyzed as continuous variables. HR, hazard ratio; CI, confidence interval; CCI, Charlson Comorbidity Index; PMI, Psoas Muscle Index; PNI, Prognostic Nutritional Index; irAE, immune-related adverse event.

**Table 5:** Multivariable Cox regression analysis for overall survival.

Variable	Adjusted HR	95% CI	*p* Value
Age, per 1-year increase	1.11	1.02–1.20	0.013
PNI, per 1-unit increase	0.86	0.81–0.93	<0.001
Antibiotic use (Yes vs. No)	3.64	1.70–7.80	<0.001

Age and PNI were analyzed as continuous variables. The Cox proportional hazards model was stratified by ICI treatment type to account for non-proportional hazards across treatment regimens. The proportional hazards assumption was satisfied for the stratified model (global Schoenfeld test, *p* = 0.37). HR, hazard ratio; CI, confidence interval; PNI, Prognostic Nutritional Index; ICI, immune checkpoint inhibitor.

## Data Availability

The datasets generated and/or analysed during the current study are not publicly available due to institutional data protection policies but are available from the corresponding author on reasonable request.
